# Immunological perspectives on the pathogenesis, diagnosis, prevention and treatment of COVID-19

**DOI:** 10.1186/s43556-020-00015-y

**Published:** 2021-01-20

**Authors:** Yanghong Ni, Aqu Alu, Hong Lei, Yang Wang, Min Wu, Xiawei Wei

**Affiliations:** 1 Laboratory of Aging Research and Cancer Drug Target, State Key Laboratory of Biotherapy, National Clinical Research Center for Geriatrics, West China Hospital, Sichuan University, Chengdu, 610041 China; 2 Department of Gynecology and Obstetrics, Development and Related Disease of Women and Children Key Laboratory of Sichuan Province, Key Laboratory of Birth Defects and Related Diseases of Women and Children, Ministry of Education, West China Second Hospital, Sichuan University, Chengdu, 610041 P. R. China; 3 Department of Biomedical Sciences, School of Medicine and Health Sciences, University of North Dakota, Grand Forks, ND 58203 USA

**Keywords:** COVID-19, Immunity, Pathogenesis, Diagnosis, Immunotherapy, Vaccine

## Abstract

Coronavirus disease 2019 (COVID-19) is an acute respiratory disease caused by severe acute respiratory syndrome coronavirus 2 (SARS-COV-2). COVID-19 can spread to the entire body and cause multiple organ failure. It is a daunting challenge to control the fast growing worldwide pandemic because effective prevention and treatment strategies are unavailable currently. Generally, the immune response of the human body triggered by viral infection is essential for the elimination of the virus. However, severe COVID-19 patients may manifest dysregulated immune responses, such as lymphopenia, lymphocyte exhaustion, exacerbated antibody response, cytokine release syndrome (CRS), etc. Understanding of these immunological characteristics may help identify better approaches for diagnosis, prognosis and treatment of COVID-19 patients. As specific anti-viral agents are notoriously difficult to develop, strategies for modulating the immune responses by either developing novel vaccines or using immunotherapy hold great promise to improve the management of SARS-CoV-2 infection.

## Introduction

As of 29 October 2020, data from the World Health Organization (WHO) reported that Coronavirus disease 2019 (COVID-19) has overspread to 219 countries with 44,002,003 confirmed cases and 1,167,988 deaths [1]. Within months of occurrence, we have had better knowledge of this novel coronavirus, named as the severe acute respiratory syndrome coronavirus 2 (SARS-CoV-2) due to its high sequence homology (94.4%) in the seven conserved replicase domains in ORF1ab to SARS-CoV-1 or SARS-CoV. Entry of SARS-CoV-2 into host cells is mediated by angiotensin converting enzyme II (ACE2), just as SARS-CoV [2]. However, the infectivity and clinical features of COVID-19 are distinct from SARS-CoV-1. SARS-CoV-2 binds to ACE2 receptor with 10-20 folds higher affinity than that of SARS-CoV-1, which facilitates the transmission of SARS-CoV-2 from human to human [3]. At present, the transmission routes have been recognized through droplet/aerosol transmission and contact transmission. Meanwhile, it is recently reported that in patients with abdominal symptoms, SARS-CoV-2 can also be detected in stool samples, suggesting a potential route of fecal-oral transmission [4]. The most common clinical manifestations of COVID-19 include fever, fatigue and dry cough, with some patients presenting atypical abdominal symptoms, such as diarrhea and nausea. Severe complications, e.g. acute respiratory distress syndrome (ARDS), shock, multiple organ failure (MDR) and death are more often in older patients with basic diseases including diabetes, hypertension and cardiovascular disease [5–7]. Supportive treatment designed for each individual and oxygen therapy are the main strategies recommended by clinical treatment protocols, with mechanical ventilation applied to patients with respiratory failure. No specific antiviral drug has been approved for COVID-19 therapy despite of the active ongoing clinical trials [8]. Despite only a few months since the first report, we have witnessed rapid advance in understanding of the immune response and regulation with COVID-19 patients. Herein, we review the immunological factors connected to SARS-COV-2 pathogenesis, as well as their implications in diagnosis, prevention, and treatment of COVID-19.

## Underlying immunological pathogenesis of COVID-19

The infectious stage of SARS-CoV-2 can be divided into three parts: 1) incubation period without symptoms; 2) non-severe symptomatic period; 3) severe symptomatic period with complications like ARDS, arrhythmia and death [9]. During the incubation and non-severe disease situations, a rapid and well-coordinated immune response is required to clear virus, preclude disease deterioration and promote recovery. However, the protective immune response is seriously impaired in patients of severe stages, causing excessive inflammation which contributes to the occurrence of fatal complications and poor prognosis [9, 10].

SARS-CoV-2 can induce alterations in the numbers and functions of both innate and adaptive immune cells, especially in severe patients. The most significant phenomenon is lymphopenia and T cell exhaustion. Increase of white blood cell, especially of neutrophils, also frequently occurs in COVID-19 patients. In addition, antibody response, mainly IgG, IgM and IgA, plays an important role in the protection of human body against the virus. Viral infection triggers the elevation of numerous pro-inflammatory cytokines which forms cytokine release syndrome (CRS), a systemic inflammatory disorder underlying the mechanism of ARDS in COVID-19 pneumonia and contributing to infection-related deaths.

### Immune cell responses

#### Reduction in lymphocyte count

**Fig. 1:**
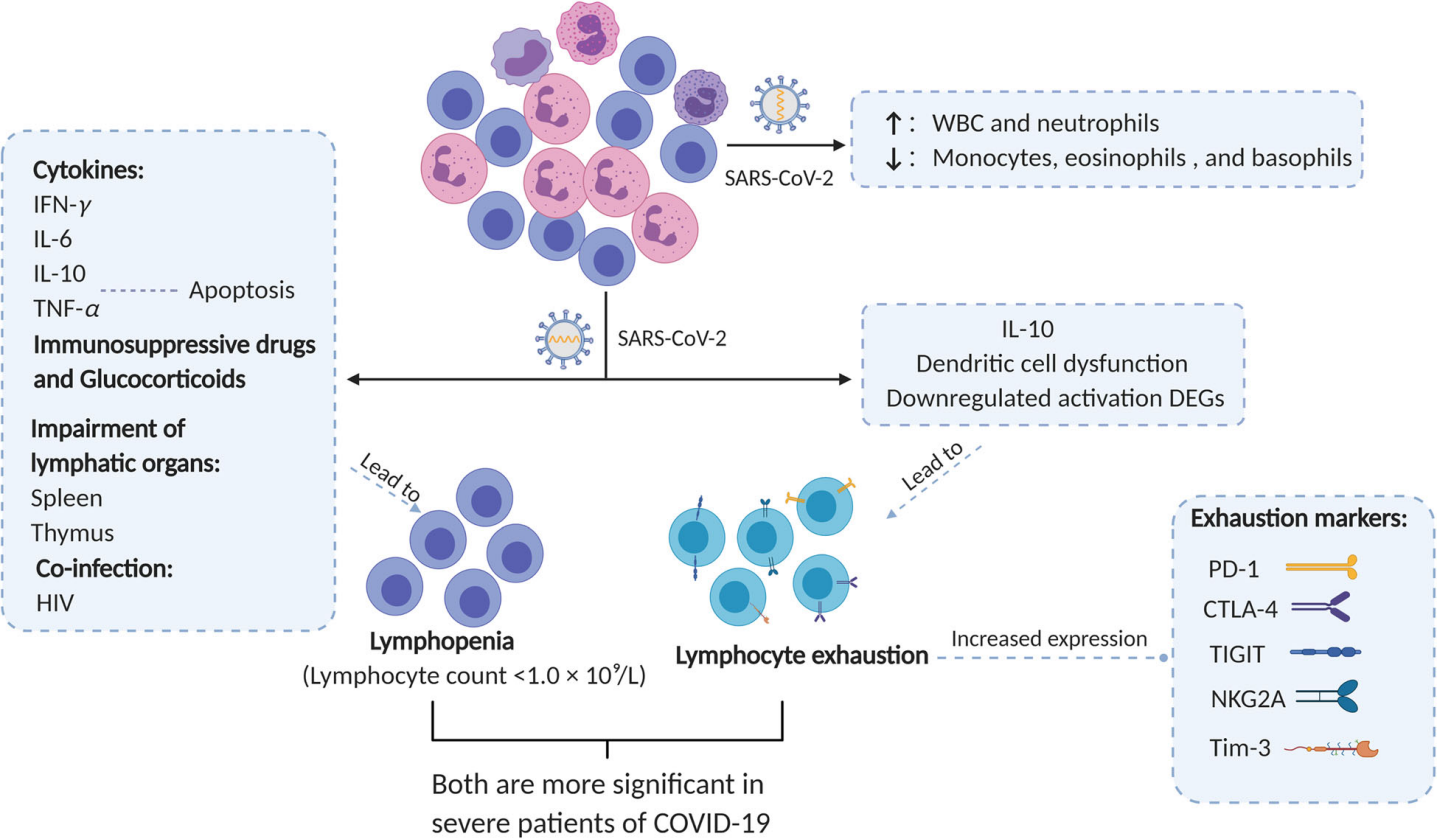
Immune responses of lymphocytes to SARS-CoV-2 infection. Lymphopenia and lymphocyte exhaustion are two important characteristics of SARS-CoV-2 infection, which are aggravated in severe patients of COVID-19 compared to milder cases. Lymphopenia may result from dysregulated cytokine release (including IFN-*γ*, IL-6, IL-10, TNF-*α*), administration of immunosuppressive drugs or glucocorticoids, impairment of lymphatic organs (i.e. spleen and thymus), and co-infection with HIV. TNF-*α* leads to lymphopenia by inducing cell apoptosis. Increased level of IL-10, virus-induced dendritic cell dysfunction and down-regulated differentially expressed genes (DEGs) involved in T cell activation are major causes of T cell exhaustion in COVID-19 patients. Lymphocyte exhaustion is featured by increased expression of exhaustion markers including PD-1, CTLA-4, NKG2A, TIGIT and Tim-3. WBC and neutrophils are continuously increased after SARS-CoV-2 infection while monocytes, eosinophils, and basophils could be decreased.

Similar to SARS-CoV-1 [11], an absolute reduction in the lymphocyte count was observed in most cases. It is reported that 63.0%-82.1% of COVID-19 patients showed decreased circulating lymphocytes in their clinical course, including up to 84.6% of severe and 44.4% of mild patients [7, 12, 13] (Fig. 1). Non-survivors showed markedly reduced lymphocyte count, suggesting the existence of immune deficiency against viral infection in COVID-19 [7, 14]. Lymphopenia (lymphocyte count <1.0 × 10^9^/L) is a common feature of patients with severe COVID-19, although not obvious in mild cases [12, 15–17]. The significantly decreased circulating lymphocytes included T cells, sometimes B cells and natural killer (NK) cells, which was more obvious in severe patients [16]. The decrease of T cells and NK cells went beyond the normal level while B cells kept in the normal range. Some severe patients even exhibited an increased percentage of B cells, probably resulting from the sharp loss of T lymphocytes [18]. These results indicated that T cell-mediated acquired immunity was preferentially damaged than B cells during SARS-CoV-2 infection. It is probably because that T cell-mediated cellular immune response is essential for direct virus eradication after virus infection whilst B cell functions in producing antibodies that neutralize virus [19]. Further analysis demonstrated that CD4^+^ and CD8^+^ T cells predominated these decreased lymphocytes [13]. As for CD4^+^ helper T (Th) cells, an increased percentage of naïve Th cells but a decreased memory Th cells were observed in severe cases. Regarding CD8^+^ T cells, a decreased percentage of cytotoxic CD8^+^ T cells (CTLs) was observed, although no difference was found in HLA-DR^+^ activated T cells [16]. In COVID-19 patients with ARDS, peripheral blood CD8:CD4 ratio was decreased compared to healthy controls, which may be a result of CD8^+^ T cell migrating to the respiratory tract [20]. COVID-19 patients also presented reduced level of regulatory T (Treg) cells including naïve Treg cells and induced Treg cells, particularly for severe cases [16, 18].

As a key feature of SARS-CoV-2 infection, lymphopenia is closely related to disease severity and mortality. In non-severe patients, the numbers of all lymphocyte subsets were normal and increased gradually during the treatment, which remained higher than the level in severe patients [21]. Activated CD8^+^ T cells in response to mild SARS-CoV-2 infection were observed to produce a large amount of granzymes A and B and perforin prior to patient recovery [22]. However, compared to mild patients, severe group showed a significant reduction of CD4^+^ and CD8^+^ T lymphocytes below normal values and lower survival rates [23]. In a retrospective study in 522 COVID-19 patients, a sharper reduction of T cells (including total T cells, CD4^+^ and CD8^+^ T cells) occurred in patients requiring intensive care unit (ICU) care *vs.* non-ICU patients [18]. A sustained decrease of lymphocytes was observed in severe patients and reached the lowest count at 4 to 6 days after disease onset [13]. Dead patients in the severe group showed a continuous fall of T lymphocytes number until death [21].

A drop in lymphocyte count could be a powerful predictor of disease progression and deterioration. Recently, Li et al. established a Time-Lymphocyte % model to classify disease and predict prognosis of COVID-19 on the basis of lymphocyte count at different time points of the disease [24]. When the numbers of CD3^+^, CD4^+^, CD8^+^ cells dropped below 900, 500 and 300 cells/μL respectively, people became susceptible to SARS-CoV-2 infection [25]. T cells decreased continuously as the disease progressed from prodromal to overtly symptomatic stages [26]. The numbers of CD3^+^ T cells (area under curve (AUC) =0.980), CD4^+^ T cells (AUC=0.972), and CD8^+^ T cells (AUC=0.933) provided a diagnostic value by identifying severe COVID-19 patients with high sensitivity and specificity, of whom the cut-off values were 575, 392, 214 cells/μL, respectively [21]. 800 cells/μL of total T cell count was regarded as a threshold for urgent intervention to non-ICU patients so as to avoid further deterioration regardless of absence in severe symptoms [26]. 559, 235, 104, 85 and 82 cells/μL of total lymphocytes, CD3^+^ T cells, CD4^+^ T cells, CD8^+^ T cells, and B cells respectively, were the warning values for in-hospital death of SARS-CoV-2 infection [27].

On the contrary, a return in lymphocyte count was essential for recovery and predicted favorable prognosis. In patients recovered from severe disease, the lymphocytes that reached its nadir within the first week would gradually return at the second week (about 15 days after treatment), increase to the approximate levels of the mild cases at the third week and become normal around 25 days after treatment [13, 21, 28]. The timeline between the recovery of lymphocyte count and the improvement of clinical course was almost concurrently. But the tendency cannot be observed in B cells and NK cells both in the dead and improved subgroups of severe patients [21]. A case report reported an increase in circulating antibody-secreting cells, follicular Th cells, CD4^+^ and CD8^+^ T cells before symptom resolution which persisted for at least 7 days after recovery, suggesting the activation of substantial anti-viral immunity in recovered patients [29]. The dynamic changes in lymphocyte number further proved the impaired cellular immunity in COVID-19 patients, which was more obvious in severe infections. Therefore, attempting to increase the number of peripheral lymphocytes may be effective in saving patients’ lives.

Up to date, it remains uncertain how SARS-CoV-2 infection induces lymphopenia. SARS-CoV-2 enters cells using ACE2 that is widely expressed on cardiopulmonary tissues and certain hematopoietic cells like monocytes and macrophages [2, 30]. Owing to low expression of ACE2 on T cell surface [2], direct viral attack of T cells *via* ACE2 receptor can hardly explain the occurrence of lymphopenia. Some researchers hypothesized that the entry of SARS-CoV-2 may be mediated by other receptors like CD147 on T cells [2, 31, 32]. Nevertheless, SARS-CoV-2 cannot replicate in T cells like MERS-CoV [33]. Additionally, since these results were not derived from primary T cell experiments, whether lymphopenia was caused by direct invasion of SARS-CoV-2 requires further evidence [34]. Other speculations concerning the underlying mechanisms of lymphopenia in COVID-19 were discussed as follows.

First, the reduction in lymphocyte count may be attributed to increased cell apoptosis. Investigators discovered a negative correlation between T cell numbers and the concentration of cytokines including interleukin-6 (IL-6), IL-10, interferon-*γ* (IFN-*γ*), and tumor necrosis factor-*α* (TNF-*α*) in COVID-19 patients [26, 27]. Highly dysregulated cytokine release might promote T lymphocyte apoptosis by activating extrinsic and intrinsic apoptosis pathways during SARS-CoV-2 infection [33]. It has been confirmed that TNF-*α* and IL-6 are important inducers of cell apoptosis [35]. TNF-*α*, particularly, can trigger T cell apoptosis by binding to its receptor, TNF receptor 1, whose expression is increased in aged T cells [33, 35]. Second, administration of glucocorticoids or immunosuppressive drugs to severe COVID-19 patients during hospitalization may promote lymphocyte depletion [15, 18]. Glucocorticoids can promote T lymphocyte loss, inhibit immune responses and delay viral clearance, accounting for the impaired T cell responses in COVID-19 patients treated with glucocorticoids [21, 36]. Two post-transplant cases of COVID-19 showed extremely low T cell counts and ended up with death even with terminated immunosuppressive agents at the moment of diagnosis [37]. The pre-existed immunosuppression might promote the immune deficiency caused by SARS-CoV-2 and deteriorate the outcome. Last, the occurrence of lymphopenia might also involve the impairment of lymphatic organs (thymus and spleen) and inhibition of metabolic molecules on lymphocytes like hyperlactic acidemia as a result of metabolic disorders [24]. It is noteworthy that coinfection with other pathogens can also interfere with the immune system and cause a synergistic damage effect. Coinfection of SARS-CoV-2 and HIV (a viral “killer” of CD4^+^ T cells) prolonged the clinical course of SARS-CoV-2 to more than 2 months. Not until two months after symptom onset was serum SARS-CoV-2-specific IgM detectable [38].

#### Lymphocyte exhaustion

Apart from reduction in cell number, the remaining lymphocytes in COVID-19 patients were functionally exhausted. Exhausted lymphocytes are a type of dysfunctional cells hallmarked by poor effector functions, increased and persistent expression of inhibitory signals, poor memory recall compared to their normal counterparts (functional effector or memory cells) [39]. The odds of multifunctional and non-functional T cells were observed to experience a significant decrease in severe patients of COVID-19, compared to healthy controls and mild patients [40]. The surviving T cells after infection expressed high levels of programmed death-1 (PD-1) and Tim-3, which were both markers of exhaustion and increased as the disease progressed [26, 41]. Severe patients exhibited higher frequency of exhausted CD8^+^ T cells that express PD-1, cytotoxic T lymphocyte antigen 4 (CTLA-4) and TIGIT (a novel immune checkpoint receptor [42]), indicating the serious damage of cellular immune response in these cases [40]. Another exhaustion marker, NKG2A, was observed to be overexpressed on CTLs and NK cells in COVID-19 patients, accompanied by decrease in their numbers. Exhausted NK cells and CTLs are defective in producing CD107a, IFN-*γ*, IL-2, granzyme B, and TNF-*α* [43]. It is noteworthy that in convalescent and recovered patients, exhaustion markers started to return to normal level, restoring the functions of CTLs and NK cells [8, 43]. These alterations suggested that T cell exhaustion-mediated immune deficiency significantly promoted disease progression.

How does lymphocytes become functionally exhausted after SARS-CoV-2 infection? It is speculated that IL-10 is an important factor [26]. An increase in the concentration of IL-10 in COVID-19 patients has been demonstrated in an array of studies [12, 13]. IL-10 is an inhibitory cytokine that suppresses cellular immune response by inhibiting proliferation and inducing exhaustion, especially in T cells. Blockade of IL-10 signaling with genetic removal or neutralizing antibody could successfully prevent T cell exhaustion and eliminate persistent viral infection in animal models [44, 45]. In addition, T cell exhaustion may result from defective activation. It is observed that in severe cases of COVID-19, differentially expressed genes (DEGs) involved in T cell activation and differentiation were downregulated, leading to T cell inactivation and damaged inflammatory response [46]. Among the DEGs, MAP2K7 and SOS1 were then upregulated after initial treatment, which can mediate T cell activation via activation of JNK pathway and ERK pathway, respectively [46–48]. SARS-CoV-2 infection-induced dendritic cell dysfunction also lead to defective activation of T cells [33, 43].

Since an increased expression of PD-1, CTLA-4, TIGIT, NKG2A and Tim-3 was responsible for lymphocyte exhaustion, downregulation or inhibition of these molecular biomarkers may hopefully reverse the functional exhaustion of anti-viral lymphocytes during SARS-CoV-2 infection and promote virus clearance at an early stage.

#### Other immune cells

SARS-CoV-2 can also affect the counts of many other immune cells, including white blood cells (WBC), leukocytes, monocytes, eosinophils, basophils etc. [12, 16] The numbers of monocytes, eosinophils (eosinopenia), and basophils could be decreased [16] or close to normal ranges [13]. However, in most cases, WBC and neutrophils were continuously increased after SARS-CoV-2 infection [7, 14, 21, 49]. Additionally, severe cases exhibited much higher counts of both WBC and neutrophils (leukocytosis) than those of moderate cases [16, 18, 50]. The numbers of WBC and neutrophils may be higher in severe group at the onset (within 3 days) but returned to the comparable level of mild cases at later phrases of disease progression [13]. The exceedingly heightened neutrophilia was responsible for viral invasion-induced CRS, leading to devastating sequelae [7, 51].

Neutrophil-to-lymphocyte ratio (NLR), with its counterpart neutrophil-to-CD8^+^ T cell ratio (N8R), is a notable marker of infection and systemic inflammation, which has been widely studied as a predictor for infectious pneumonia [52, 53]. Increase in NLR and N8R was frequently present in COVID-19 patients, which was usually associated with higher disease severity and poor clinical outcome [7, 16, 49, 51]. Patients with high levels of NLR showed elevated pro-inflammatory cytokines including IL-2, IL-6 and IL-10 in comparison with low NLR patients [49]. NLR and N8R were powerful prognostic factors for prognosis of severe COVID-19, with AUC of 0.93 and 0.94 respectively [13]. Therefore, supervision of NLR and lymphocytes is useful for early screening and diagnosis, as well as identification of severe cases and improved management of COVID-19 patients.

### Antibody responses

Among SARS-CoV-2 infection, humoral immune response was evoked by producing neutralizing antibodies (NAbs) that could block the receptor binding-mediated viral entry into host cells. The Spike (S) protein, including its S1, S2 and receptor binding domain (RBD) regions, is highly immunogenic and can serve as a target of many NAbs [54, 55]. Nucleocapsid (N) protein-specific antibody response was also observed in the sera of COVID-19 patients but showed no neutralizing activity [55, 56]. These antibodies cannot cross-neutralize SARS-CoV-1, suggesting the diverse epitopes and immunogenicity between these two viruses [55, 57]. An elevation was observed in the numbers of activated CD4^+^ cells, antibody-secreting cells, as well as IgM and IgG titers in the blood of mild COVID-19 patients which persisted for at least 7 days after recovery [22, 29]. CD4^+^ T cells facilitated the generation of virus-specific antibody by activating B cells [18]. Plasma cells were dramatically activated in the bronchoalveolar lavage specimen of a COVID-19 patient requiring extracorporeal membrane oxygenation [58]. Older age was considered as a predictor for poor prognosis [59], but recovered older patients in a cohort study of 175 COVID-19 cases showed a higher S protein-specific NAb level and activity than the younger ones. The decrease in lymphocyte count in elderly patients was negatively correlated to NAb titers [18]. Therefore, there may be an adaptive enhancement of humoral response for compensation of the impaired cellular response, in order to help the older clear the virus effectively and recover from the disease.

IgM, IgG and IgA are major antibody types showing a steady increase and strong activity, while IgG1 and IgG3 dominate the IgG subtypes [2, 22, 56, 60–62]. Seroconversion of S-protein specific IgG and IgM was reported to occur by day 7 to 19 after symptom onset [57, 63]. The median time for detection of various antibodies for SARS-CoV-2 was about 1 to 2 weeks [64]. Up to 100% of patients were tested positive for IgG and approximately 94.1% for IgM [57]. Of note, titers of NAbs in COVID-19 patients, including the convalescent, were widely variable, ranging from undetectable (<30) to 21567 [55–57, 65]. IgM presented a decrease in 2 to 3 weeks after clinical symptoms onset [2, 57]. In the follow-up of discharged patients, IgG levels were much higher than IgM, suggesting the long term (at least for two weeks after discharge) immunity against viral infection by producing IgG antibodies [56]. Therefore, IgM antibodies provided protective immunity against SARS-CoV-2 at the early stage whereas IgG antibodies were the main components of enduring immunity.

The complement system also contributed to prognosis of COVID-19 pneumonia. Severe patients had higher concentration of complement C3 than non-severe group [16, 21]. Inhibition of complement C3 may alleviate the proinflammation response and acute lung injury, providing an alternative treatment strategy for SARS-CoV-2 infection [21]. Complement C3 inhibitor (AMY-101, NCT04395456) and complement C5 inhibitor (Zilucoplan, NCT04382755; Eculizumab, NCT04288713, NCT04355494) are currently under clinical trials to evaluate their efficacy in the management of COVID-19 patients, particularly those with ARDS.

### Cytokine release syndrome

**Fig. 2:**
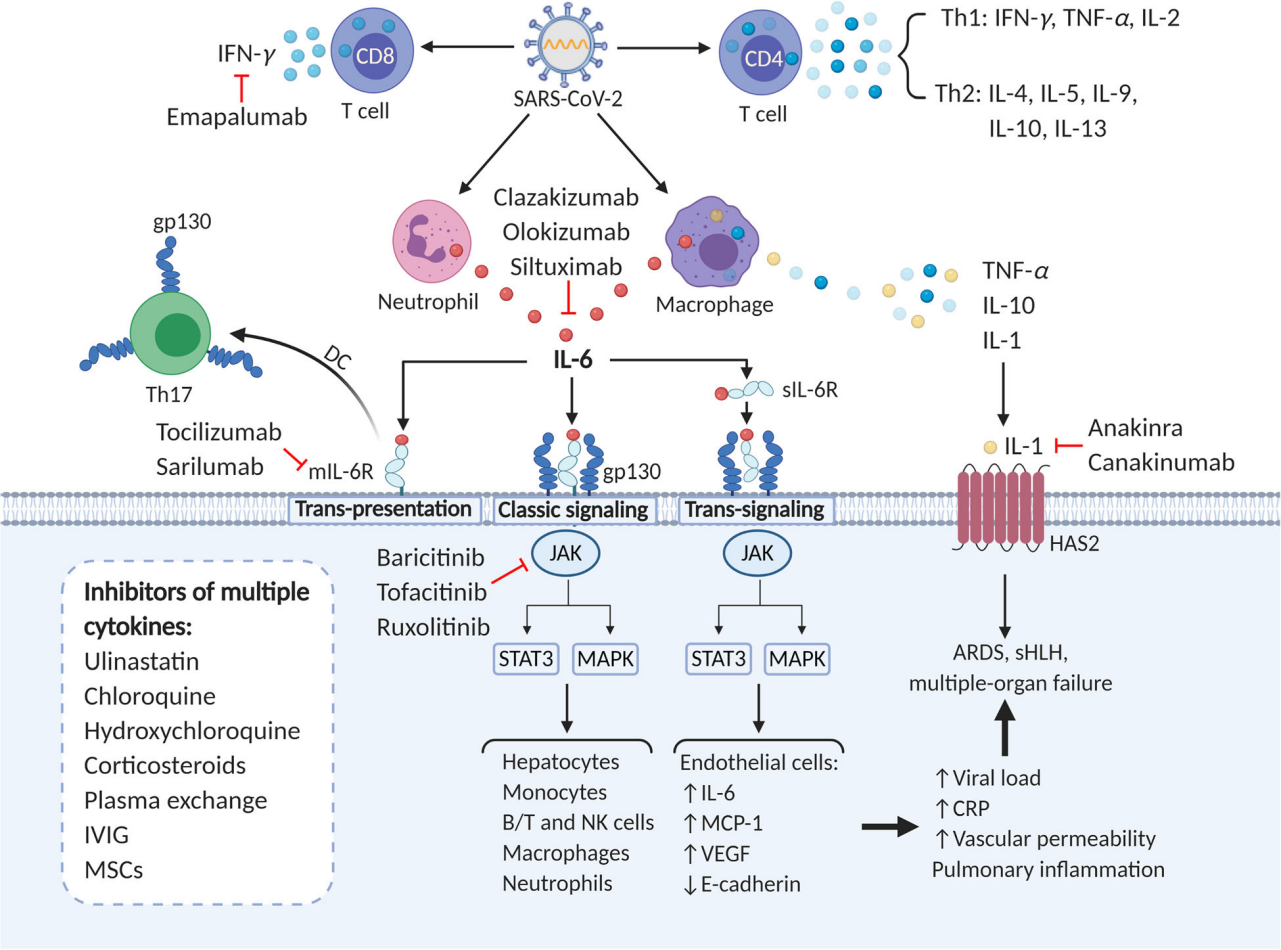
Cytokine release syndrome in COVID-19 and relevant therapeutic options. CRS is commonly observed in severe COVID-19 patients involving an excessive production of various inflammatory cytokines. SARS-CoV-2 infection initiated increased secretion of both Th1 cell cytokines (IL-2, IFN-*γ*, TNF-*α*) and Th2 cell cytokines (IL-4, IL-5, IL-9, IL-10, IL-13). CD8^+^ T cells may predominantly produce IFN-*γ*. Activation of macrophages and neutrophils may also account for the generation of multiple cytokines, such as IL-6, IL-1, IL-10, TNF-*α*, etc. among which IL-6 is the predominant component in causing CRS. IL-6 can induce CRS through three different signaling pathways: classic signaling, trans-signaling and IL-6 trans-presentation. Three pathways all function with the formation of a hexameric complex including IL-6R (both mIL-6R and sIL-6R) and gp130, leading to the activation of downstream intracellular JAK- MAPK and JAK- STAT3 signaling pathways. Later on, cells expressing mIL-6R including monocytes, macrophages, etc. and endothelial cells that do not express mIL-6R are overactivated to accelerate secretion of cytokines, which cause increased viral load, CRP and permeabilization with subsequent ARDS, sHLH and multiple-organ failure. Apart from IL-6, IL-1 may also drive the occurrence of ARDS in COVID-19 patients by inducing HAS2. To ameliorate CRS, we can use inhibitors of multiple cytokines including chloroquine and corticosteroids, or use specific inhibitors targeting IL-6, IL-6R, IL-1, JAK and IFN-*γ*. mIL-6R: membrane IL-6 receptor; sIL-6R: soluble IL-6 receptor; DC: dendritic cell; HAS2: hyaluronan-synthase-2; IVIG: intravenous immunoglobulin; MSCs: mesenchymal stem cells.

Accumulating evidence indicated that a subgroup of severe COVID-19 patients underwent CRS, which is a systemic inflammatory disorder induced by cytokine storm (Fig. 2). It is characterized by the elevation in serum levels of multiple pro-inflammatory cytokines including IL-6, IL-8, IL-10, IFN-*γ*, TNF-*α*, granulocyte-colony stimulating factor (G-CSF) etc. [66, 67] In SARS-CoV and MERS-CoV, CRS was discovered to be the major cause of fatality, probably by inducing ARDS and secondary hemophagocytic lymphohistiocytosis (sHLH) [68, 69]. sHLH is hallmarked by CRS and multiorgan failure with high concentrations of ferritin and additional cytokines such as IL-18, IFN-*γ* inducible protein 10 (IP10), monocyte chemoattractant protein 1 (MCP1), and macrophage inflammatory protein 1*β* (MIP1*β*) [70, 71]. In most severe COVID-19 patients, an excessive production of various inflammatory cytokines and chemokines were observed, including IL-1*β*, IL-2, IL-4, IL-6, IL-7, IL-8, IL-9, IL-10, IL-17, TNF-*α*, IFN-*γ*, IP10, MIP1*α*, MCP1, MCP3, G-CSF and granulocyte-macrophage colony-stimulating factor (GM-CSF) [8, 12–14, 16, 18, 26, 72–75]. Unlike SARS-CoV-1 infection [76], SARS-CoV-2 infection increased secretion of both Th1 (e.g. IFN-*γ*, IP10, and MCP1) and Th2 cell cytokines (e.g. IL-4 and IL-10) [12].

Increased inflammatory factors contributed to disease deterioration, which makes CRS a critical indicator of disease severity in COVID-19 pneumonia. Severe patients possess higher plasma levels of these cytokines than the moderate. It is reported that IL-6 in critically ill patients was almost 10 times higher than other patients [77]. And elevated IL-6 concentration was closely related to the identification of detectable serum SARS-CoV-2 viral load (RNAemia, R=0.902) which can only be detected in critically ill patients [77]. The elevation of IL-6, IL-10, IFN-*γ*, and TNF-*α* levels may be the reason for profound T cell loss, making lymphopenia another hallmark of serious illness [12, 13, 18]. Besides, severe patients presented more significant fluctuations in the serum concentrations of these cytokines than the mild group. Cytokine levels of severe cases peaked on 3 to 6 days after disease onset which is the moment that T cells dropped to its lowest number, whereas IL-6 and IL-10 levels presented sustained elevations. In patients recovered from the severe disease group, a gradual decline of IL-2, IL-4, IL-6, IL-10, IFN-*γ* and TNF-*α* concentrations in the serum was observed, synchronizing with a steady increase in lymphocyte count [13]. Therefore, monitoring the changes in cytokine levels can help predict a deterioration or improvement of the disease.

These increased cytokines induced vascular hyperpermeability, pulmonary inflammation, extensive lung damage and multiorgan failure in COVID-19 [12, 72]. Nonspecific inflammatory cell infiltration and local excessive cytokine generation account for the pulmonary and interstitial tissue damage of ARDS [78, 79]. Increased vascular endothelial growth factor (VEGF) secretion and reduced E-cadherin expression triggered by IL-6 participated in the vascular permeability and leakage, contributing to pulmonary dysfunction of ARDS [69]. Postmortem examination of non-survivors showed desquamation of pneumocytes, pulmonary edema, hyaline membrane formation, as well as interstitial infiltration of mononuclear inflammatory lymphocytes, similar to SARS, and MERS [15]. Of note, CRS in SARS-CoV-2 infection also had an impact on neuroendocrine system to release glucocorticoids or other peptides, damaging the immune system [8]. These results indicated that CRS-induced ARDS and sHLH existed in the severe stage of SARS-CoV-2 infection. Since ARDS and extrapulmonary multiple-organ failure were the decisive factors for disease exacerbation and major cause of death, a better understanding of the underlying mechanisms of CRS could hopefully guide the exploration of strategies to terminate the pathological process.

The cellular sources and underlying mechanisms of CRS in COVID-19 have not been fully clarified yet. Previous studies demonstrated that T cells, macrophages and monocytes are the main sources [80, 81]. Despite the observed lymphopenia in most severe cases, some studies indicated that overactivation of T cells occurred in COVID-19 patients before the initiation of lymphocyte reduction and exhaustion [15]. Therefore, it is speculated that in the early stage of SARS-CoV-2 infection, overactivated lymphocytes may produce tremendous amount of cytokines [82] which can in turn inhibit lymphocytes themselves. CD8^+^ T cells predominantly produced IFN-*γ*, while CD4^+^ T cells secreted typical Th1 (IL-2, IFN-*γ*, TNF-*α*) and Th2 (IL-4, IL-5, IL-9, IL-10, IL-13) cytokines [12, 20, 83]. But IL-6 was not originated from T cells [20]. Macrophage and neutrophil activation in COVID-19 also contributed to the excessive cytokine release [9, 16]. Single cell RNA sequencing of bronchoalveolar immune cells from COVID-19 patients showed that pro-inflammatory macrophages were abundant in the bronchoalveolar lavage fluid from severe patients, contributing to higher levels of inflammatory cytokines [84]. By infecting transgenic mice that express human ACE2 with SARS-CoV-2, researchers found that the alveolar interstitium became infiltrated with numerous macrophages and lymphocytes. Plenty of macrophages also accumulated in alveolar cavities [85] Most of the immune cells were able to produce IL-10 which could induce immune suppression and prevent the release of various cytokines by different cells [86]. These results indicated that macrophages, lymphocytes and neutrophils all contributed to the secretion of excessive cytokines after SARS-CoV-2 infection.

Viral infection induced CRS by activating NF-*κ*B pathway through pattern recognition receptors and angiotensin 2 (AngII)-angiotensin receptor type 1 (AT1R) axis [87]. Hyperactivation of NF-*κ*B pathway promoted the production of numerous pro-inflammatory cytokines, including IL-6 [88]. Signal transducer and activator of transcription 3 (STAT3) is necessary for full activation of the NF-*κ*B pathway while IL-6 is the main trigger of STAT3 activation [89]. Contrarily, ACE2 is an important membrane protein and inactivator of AngII. In SARS-CoV infection, ACE2 expressed on the surface of target cells was occupied by the virus and endocytosed, leading to a reduction of ACE2 and ensuing increase of serum AngII [90]. However, direct proof on the existence of NF-*κ*B pathway and AngII- AT1R axis in SARS-CoV-2 is still insufficient. Activation of coagulation pathways during the immune response may also promote the occurrence of CRS, as D-dimer was found to be abnormally high in severe COVID-19 pneumonia [8, 72].

IL-6 is the most important leading cause of CRS among all these increased cytokines [66, 82]. IL-6 is a multifunctional cytokine that is essential for immunity, tissue regeneration, and metabolism. Proper amount of IL-6 protect individuals against pathogen infection and tissue injury, whereas excessive IL-6 production results in pathological disorders [91]. IL-6 binds to its receptor IL-6R to trigger its signaling cascades, involving the activation of a signal transducer, gp130 [92]. Three IL-6 signaling pathways have been determined: classic signaling, trans-signaling, and IL-6 trans-presentation. In classic signaling, IL-6 binds to trans-membrane IL-6R (mIL-6R) and gp130 on IL-6R-expressed cells like hepatocytes, monocytes, and lymphocytes, leading to pleiotropic effects on multiple immune cells including B cells, T cells, NK cells, macrophages and neutrophils. In trans-signaling, IL-6 binds to soluble IL-6R (sIL-6R), forming an IL-6-sIL-6R complex which then binds to membrane-bound gp130 on IL-6R deficient cells such as endothelial cells, evoking enhanced secretion of additional IL-6, MCP1, VEGF and reduced expression of E-cadherin. In trans-presentation, IL-6-mIL-6R complex was presented by specialized dendritic cells on their membrane to Th17 cells expressing gp130, which explained how excessive IL-6 induced overactivation of Th17 cells in COVID-19 patients. These pathways all function with the formation of a hexameric complex including gp130, which leads to the activation of downstream intracellular the Janus kinase (JAK)-mitogen-activated protein (MAP) kinase (MAPK) and JAK- STAT3 signaling pathways [15, 91, 93, 94].

The expression of most inflammatory cytokines was observed to peak after respiratory function nadir, apart from the expression of IL-1 and IL-1R, indicating that IL-1 signaling pathway may drive the pathogenesis of COVID-19 at the early stage [95]. Moreover, it is speculated that neutrophil extracellular traps (NETs)-IL-1*β* loop was activated in severe COVID-19 patients, amplifying the generation of NETs and IL-1*β* that accelerated the progress of CRS [96]. IL-1 and TNF were strong inducers of hyaluronan-synthase-2, an important factor for the pathological changes of ARDS and a potential cause of COVID-19 fatality [9, 97]. Hence, IL-1 was another important inducer of the pathological manifestations in COVID-19. Inhibiting IL-1 pathway could potentially help control CRS and prevent the respiratory dysfunction.

In contrast, type-I IFN (IFN-I, including IFN-*α* and *β*) and type-III IFN (IFN-III) activity were significantly impaired in severe COVID-19 patients. IFN-I and IFN-III are critical for antiviral immunity. However, SARS-CoV-2 infection drove a reduced antiviral transcriptional response including IFN-I and IFN-III, followed by downregulated IFN-stimulated genes (ISGs) [98, 99]. Plasma levels of both mRNA and protein of IFN-*α*2 and IFN-*β,* as well as their activity were lower in COVID-19 patients, causing increased plasma viral load in severe cases [99]. It has been proven that SARS-CoV-2 was substantially attenuated in the context of IFN-I pretreatment [100]. Addition of IFN-I post-infection dramatically inhibited virus replication *in vitro* [98, 100] and increased ISGs score basing on mean expression of six ISGs defining a IFN-I signature [99]. These data indicated that SARS-CoV-2 infection didn’t affect the susceptibility to IFN-I, making IFN-I supplement a promising therapy.

We can conclude that dysregulated immunity at least plays an irreplaceable role in the pathogenesis of SARS-CoV-2 infection. If the immune response in incubation period and non-severe stage is effective, the host can efficiently eradicate the virus and recover from the infection. However, patients with impaired immunity mainly characterized by lymphopenia, lymphocyte exhaustion, increased NLR and CRS are more prone to develop into severe stage. It was reported that the mortality of severe COVID-19 patients was 61.5% [101]. Therefore, early diagnosis and intervention of underlying severe patients are necessary to reduce the mortality. For effective management of SARS-CoV-2 infection, we are supposed to boost immunity in non-severe patients but inhibit the hyperinflammation in severe ones.

## Immunological diagnosis of COVID-19

At present, viral nucleic acid detection of SARS-CoV-2 has been considered to be the gold-standard in diagnosis. However, it has limitations that inappropriate collection, storage, and transportation of samples as well as the improper method of real time reverse transcriptase quantitative polymerase chain reaction (RT-qPCR) may lead to false negative results [102, 103]. Therefore, it is of vital importance to develop a complementary method to diagnose patients and differentiate suspected patients fast and accurately.

Up to date, there are four most popular methods to test antibodies titers in patients’ plasm or serum, namely chemiluminescence immunoassay analysis (CLIA), enzyme-linked immunosorbent assay (ELISA), lateral flow immunoassay (LIFA) and colloidal gold immunochromatographic assay (GICA).

CLIA is one of the most advanced technologies with high sensitivity, strong specificity and wide detection range. Huang’s team revealed the dynamic changes of SARS-CoV-2 antibodies in COVID-19 patients using magnetic chemiluminescence enzyme immunoassay (MCLIA). They found the positive rate of IgG reached 100% at 17-20 days after onset, and that of IgM was as high as 94.1% at about 20-22 days after onset among 285 patients. The median time of IgG and IgM serum conversion was 13 days after onset among 26 cases. Importantly, they proved the clinical value of antibody detection in differential diagnosis of suspected cases of COVID-19 and screening of close contacts [57]. Hou et al. enrolled 338 COVID-19 patients (64 mild cases, 199 severe cases, 75 critical cases) confirmed by RT-PCR and tested their IgG and IgM titers via CLIA. Interestingly, no significant difference was found in positive rates of IgM and IgG between mild, severe and critical groups. However, they revealed higher IgM levels and lower IgG levels in critical cases than those in mild groups, which may attribute to strong virus activity and/or compromised immune response. Therefore, the result indicates the value of quantitative detection of SARS-CoV-2 antibodies in assessing the prognosis and severity of COVID-19 [104]. A retrospective study conducted by Jin et al. also displayed the diagnostic value of serological test for COVID-19 diagnosis, which contained 43 laboratory confirmed COVID-19 patients and 33 controls. The sensitivity and specificity of IgG were 88.9% and 90.9%, and that of IgM were 48.1% and 100%, respectively [105]. Qu et al. detected the serological responses to SARS-CoV-2 N and S glycoprotein, concluded that the window phase of antibody production was between 17 and 23 days after illness onset [106]. Similarly, Zhong et al. analyzed the receiver operating characteristics (ROC) curve of IgG and IgM by testing S and N antigens, concluded that the optimal cut off value for IgG and IgM were 0.199 and 0.230, respectively [107]. Additionally, viral envelope protein and N antigen from 112 COVID-19 patients were also studied. The results showed the dynamic change of antibodies level with disease progression. Briefly, IgM was produced within one week after onset and last for one month, while IgG appeared 10 days after onset and last for a longer time [108]. Interestingly, Zeng et al. evaluated the difference of IgG antibody levels in serum between sexes. They enrolled 331 COVID-19 patients (127 males and 204 females), and found a relatively higher level of IgG in female patients at the early or severe stage of disease compared to male patients [109]. It has been proved that no maternal-infant transmission of SARS-CoV-2 based on nucleic acid test [110]. However, in a study contained 6 mothers diagnosed with COVID-19, SARS-CoV-2 specific IgG and IgM were detected in the neonatal blood. IgG can be passively transferred across the placenta from mother to fetus, while how IgM appeared remains unknown [111]. Given the broad use of SARS CoV-2 antibodies IgM and IgG CLIA kits from Shenzhen YHLO Biotech Company, China. Infantino et al. made an assessment of its diagnostic accuracy based on Italian population, and proved the accuracy and efficiency of CLIA kits [112]. Padoan et al. assessed the analytical performances of MAGLUMI 2000 Plus CLIA assay (Snibe, Shenzhen, China), obtaining satisfactory repeatability and precision [113].

ELISA, a quantitative method based on antigen-antibody reaction by using an enzyme-linked conjugate and enzyme substrate, is used for routine measurements of molecular concentration [114]. Zhao et al. developed a SARS-CoV-2 S1 serology ELISA kit with overall accuracy at 97.3% through CHO cell expressed SARS-CoV-2 S1 protein. With enrolling 412 controls and 69 patients, the sensitivity and specificity were 97.1% and 97.5%, respectively [115]. Xiang et al. conducted a diagnostic test of ELISA-based IgM, IgG for COVID-19. They concluded that seroconversion of IgG and IgM appeared as early as the fourth day after onset, and that the consistency rate of IgM and IgG are 88.1% and 88.9% [61]. It has been proved that S antigen based IgM and IgG results have stronger sensitivity and specificity compared with N antigen by detecting N and S specific IgM and IgG (N-IgM, N-IgG, S-IgM, S-IgG) simultaneously among 214 patients [116]. In addition, the levels of S-IgG in ICU patients (n=11) were significantly higher than non-ICU patients (n=27), and a negative relationship between S-IgG and C-reactive protein (CRP) was disclosed among ICU patients [64]. In a serological test for healthcare workers based on S antigen, 5 of 316 (1.6 %) were found to be IgG positive whose PCR results were negative [117].

LFIA, also called the immunochromatographic (IC) test, is an advanced point-of-care test with the advantages of simplicity, rapidity and economy [118]. Li et al. developed a kind of LFIA test kit for the simultaneous detection of IgM and IgG in blood sample within 15 minutes, with sensitivity of 88.66% and specificity of 90.63% among 397 patients and 128 controls [119]. Sood et al. made a survey about the seroprevalence of SARS-CoV-2–specific antibodies among adults in Los Angeles using LFIA. 35 of 863 individuals were found to be antibodies positive, and the weighted prevalence was 4.65% [120]. In addition, a recent survey about the seroprevalence of SARS-CoV-2 specific IgM and IgG in Wuhan among 17368 individuals using MCLIA indicated that the seropositivity ranged from 3.2% to 3.8% in different subcohorts. With the increase of the distance to Wuhan, the seropositivity decreased gradually [121]. It is claimed that the positive rate of SARS-CoV-2 specific antibodies in RT-PCR confirmed cancer patients is significantly lower than that in healthcare workers [122]. Lee et al. monitored the dynamic changes of anti-SARS-CoV-2 IgM and IgG antibodies within 14 COVID-19 patients. They found that the window period time for IgM detection was 5-42 days after onset, while IgG was persistently detectable after seroconversion [123]. The results of the diagnostic test performed by Spicuzza et al. who enrolled 30 patients and 7 controls showed that the sensitivity and specificity of IC test were 83% and 93%, respectively [124]. However, Imai et al. drew a conclusion that the sensitivity of IC is low in the early stage of infection by recruiting 112 patients and 48 cases. So it is not recommended to use IC assay alone for preliminary diagnosis of covid-19 [125]. Finally, Demey et al. compared the antibodies detection efficiency of 4 IC tests provided by Biotime Biotechnology Co, Autobio Diagnostics Co, ISIA BIO-Technology Co and Biolidics, respectively. All of the tests displayed a sensitivity of 60% to 80% on day 10 and 100% on day 15. Thus the value of IC test in the detection of anti-SARS-CoV-2 antibodies has been validated [126].

GICA is a qualitative immunolabeling technology with colloidal gold as tracer. In a prospective cohort containing 150 patients with fever or respiratory symptoms, the sensitivity and specificity of GICA were 71.1% and 96.2%, respectively, with PCR results as golden standard. In subgroup analysis, the sensitivity was affected by period from symptom onset and clinical severity [127]. It was found that SARS-CoV-2 specific IgG was still detectable at the 50^th^ day after infection [128].

Though serological test is faster, more convenient and relatively cheaper than molecular detection, its limitations must be recognized as well [129]. Lv. et al. indicated that the cross-reactivity in antibody binding to the S protein between SARS-CoV-1 and SAR-CoV-2 is common [130]. Moreover, cross reactivity of S protein between SARS-CoV-2 and MERS-CoV has also been tested [131]. Therefore, it is necessary to make accurate diagnosis based on the combination detection of nucleic acid and antibodies [132]. Zhao et al. concluded that combining detection of viral RNA and antibody improved the diagnostic efficiency notably, even within one week after onset [133]. Yong et al. indicated that the viral RNA was abundant enough for RT-PCR, while the sensitivity of antibody detection exceeds that of RNA detection from the 8^th^ day after onset. Moreover, seroconversion appeared after 7 days in most of the cases whose viral RNA loading was not detectable at the early stage [134].

In summary, nucleic acid testing provides direct evidence of virus infection, while serological antibodies detection is an indirect method. Combination of the two pathways can improve sensitivity of pathogenic diagnosis for COVID-19. Detection of viral RNA by RT-PCR is still the current gold standard for diagnosis. Next, it should be noticed the cross reaction of SARS-CoV-2 with SARS-CoV-1 and MERS-CoV in utilizing antibody detection. Finally, antibodies detection is suggested to be the complementary method to nucleic acid testing, used for screen of suspected cases and population in close contact, for diagnosis of convalescence patients and for seroepidemiological survey.

In addition to antibody testing, the significance of blood routine examination and blood biochemical analysis have also been studied. First, it was proved that CD3^+^T, CD4^+^T, CD8^+^T cells and NK cells were markedly reduced among COVID-19 patients [135, 136]. The SARS-CoV-2 RNA load was negatively correlated with lymphocyte count, CD4 ^+^ and CD8 ^+^ T lymphocyte count (P < 0.001) [137]. Second, elevated NLR was found to be an independent risk factor for severe COVID-19 patients, considered as an early warning signal of a poor clinical outcome [138, 139]. Additionally, the concentration of CRP increased significantly in the early stage, predicting early severe COVID-19 [140]. Third, distinctly differences in levels of D-dimer, IL-2R, IL-6, IL-8 between the mild patients and severe patients have been disclosed as well [137, 141]. In conclusion, it is of great value to make auxiliary diagnosis in combination with immunocytes changes and other serum indexes such as D-dimer, cytokines, CRP, etc.

## Vaccine development of COVID-19

**Fig. 3:**
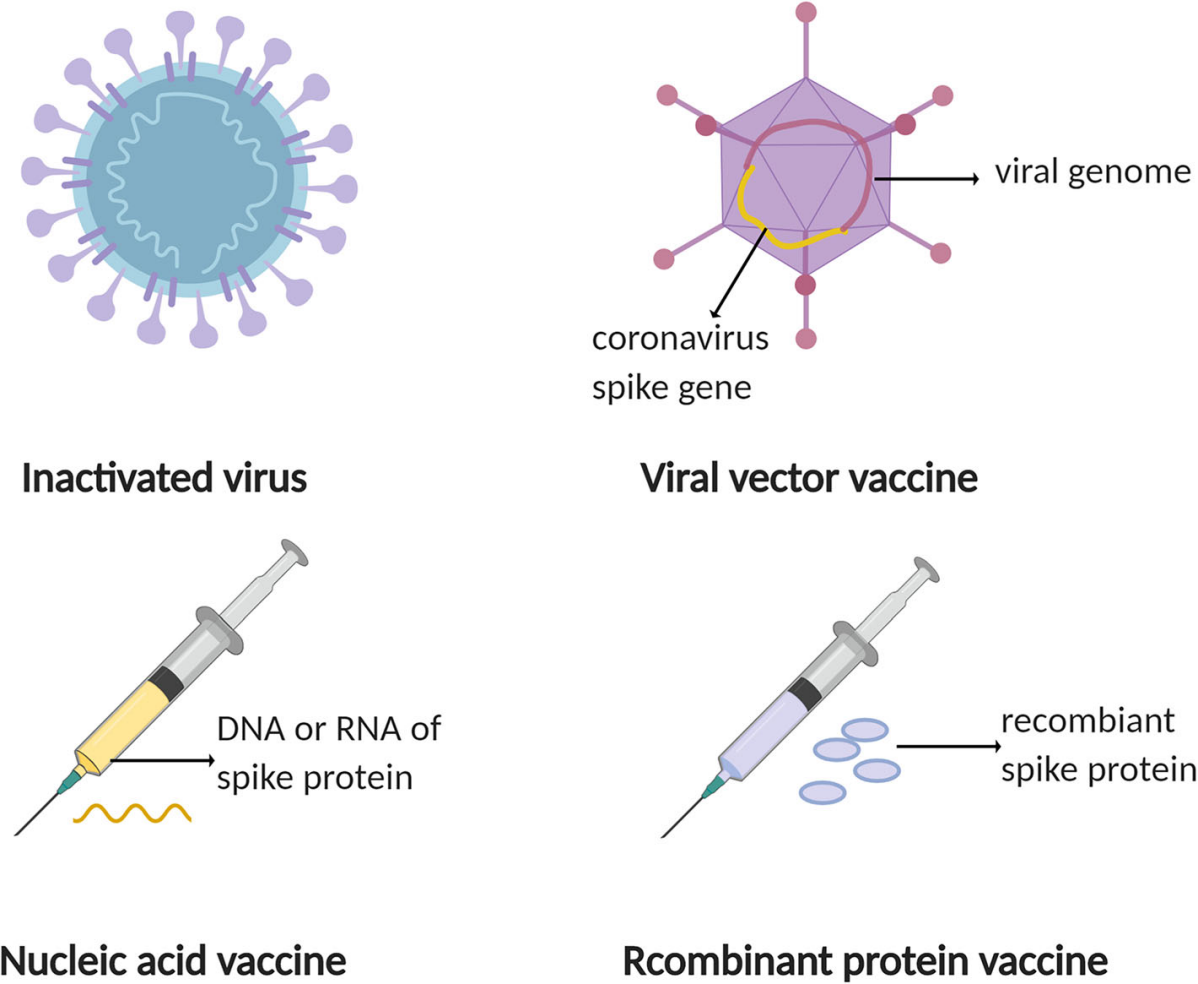
Strategies for development of vaccines. The spike glycoprotein is a key target for vaccine design due to its indispensable function in recognition of host cells and mediating fusion of the virus with cell membrane. Inactivated vaccine: to make sure the safety of inactivated vaccines, the virus was treated by chemicals, such as formaldehyde or heat. Viral-vector vaccine: The adenovirus is usually a preferable choice for vaccine vector. The vector was genetically modified to produce spike protein of SARS-CoV-2. Of note, the virulence of viral vector was weakened so it would not cause disease. Nucleic acid vaccine: Following the injection of DNA or RNA of spike protein, nucleic acids were inserted into human cells, synthesizing copies of spike protein. Recombinant protein vaccine: Genetic segments of target antigen were selected and recombined into an expression system. The recombinant spike protein was prepared *in vitro* and purified for further use.

Vaccine is the most effective measurements to control and prevent the spread of infectious disease by establishing immune defense system within human bodies. The S glycoprotein is a key target for vaccine design due to its indispensable function in recognition of host cells and mediating fusion of virus and cell membrane [3]. Totally, there are mainly four kinds of vaccines, including live attenuated vaccine and inactivated vaccine, virus vector vaccine, nucleic acid vaccine and recombinant protein vaccine (Fig. 3).

Inactivated virus vaccine, the most traditional one, has decades of application experience. It stimulates the immune system to produce antibodies by injecting inactivated viruses into humans. Gao et al. developed a kind of purified chemically inactivated vaccine named PiCoVacc by isolating SARS-CoV-2 strains from the bronchoalveolar lavage fluid samples of 11 patients. It is pointed out that the candidate vaccine successfully induced the viral specific NAbs in BALB/c mice, Wistar rats and rhesus macaques [142]. A relevant phase 1/2 clinical trial involving 744 volunteers is currently underway. In addition, Chinese scientists developed another SARS-CoV-2 inactivated vaccine candidate BBIBP-CorV. With the characteristics of highly efficient proliferation and high genetic stability, BBIBP-CorV elicited potent humoral immune response in multiple animal models including cynomolgus monkeys and rhesus macaques. Two doses of immunization (2ug/dose) were capable to protect rhesus macaques from SARS-COV-2 challenge, without detectable side effects. Phase 1/2 clinical trials of BBIBP-CorV have been initiated recently [143].

Adenovirus usually causes mild infection and is suitable for vaccine vector. Notably, the Lancet published the results of the first, in human, phase 1 clinical trial of COVID-19 vaccine on May 22. The trial evaluated the safety, immunogenicity of different doses of a recombinant adenovirus type-5 vectored COVID-19 vaccine in 108 healthy adults aged 18-60. The results showed that the candidate vaccine was well tolerated and no serious adverse events were reports within 28 days after vaccination. The most common adverse reactions were mild pain (54%, 58/108), fever (46%, 50/108), fatigue (44%, 47/108), headache (39%, 42/108) and muscle pain (17%, 18/108). Within two weeks after vaccination, antibodies were detectable in all three groups and also a rapid T cell response was induced in most of the subjects [144, 145]. Subsequently, a randomized, double-blind, placebo-controlled, phase 2 trial was conducted to further assess the immunogenicity and safety of this recombinant adenovirus type-5 vectored COVID-19 vaccine in 508 participants (50% male; mean age 39.7 years). The results suggested that a single dose of 5×10^10^ viral particles was safe, which caused only mild adverse reactions and no severe adverse events were observed. Besides, a single dose of vaccination elicited significant humoral and cellular immune response on the 28^th^ day. The seroconversion was 97% , and the RBD-specific ELISA antibodies peaked at 571.0, with geometric mean titers of 18.3 in the group receiving the dose of 5×10^10^ viral particles (n=129) [146]. Van et al. designed a ChAdOx1-vectored vaccine platform encoding the S protein of SARS-CoV-2 and evaluated its protective efficacy in mice and rhesus macaques. Strong humoral and cellular immune response were elicited after a single vaccination, which was not dominated by Th2 lymphocytes. Though the vaccine was able to prevent rhesus macaques from developing pneumonia after SARS-CoV-2 challenge, it needed to be highlighted that no difference in viral load in nose swabs was observed between vaccinated and unvaccinated animals, indicating that the vaccinated group was infected as well even if they do not have symptoms [147]. Furthermore, the evidences of its safety and efficacy based on a phase 1/2, single-blind, randomized controlled clinical trial have been studied. 543 participants received ChAdOx1 nCoV-19 at a dose of 5 × 10^10^ viral particles, with a meningococcal conjugate vaccine as control (n=534). The dose was proven to be safe and no serious adverse events were identified. After a second vaccination, both NAbs and immune response were induced potently among all receivers [148]. Hassan et al. developed a chimpanzee adenovirus- based vaccine encoding a pre-fusion stabilized S protein (ChAd-SARS-CoV-2-S) and they found that both intramuscular injection and intranasal administration stimulated strong humoral and cellular immune response. However, only intranasal administration completely protect mice from SARS-CoV-2 challenge in both the upper and lower respiratory tracts [149].

Nucleic acid vaccine, also known as gene vaccine, is designed to immunize the body with foreign gene (DNA or RNA) encoding a specific antigen. As a novel vaccine, it has not been utilized widely. Based on their prior experience of developing an engineered DNA vaccine targeting the S protein of MERS coronavirus, Smith et al. generated a synthetic DNA vaccine targeting the S protein of SARS-CoV-2, named INO-4800. The results from the animal experiments showed that antigen specific humoral and T cell responses were observed after immunizing mice and guinea pigs with INO-4800. Specific NAbs which enabled to block the binding of S protein with ACE2 receptor were detectable as well. This study supports the further assessment of INO-4800 in clinical trials [150]. The team of Dan H. Barouch developed six different variants of the SARS-CoV-2 S gene and evaluated their immunogenicity in 35 rhesus macaques. They discovered that the neutralizing antibody tiers in vaccinated animals were comparable to those found in convalescent patients. The vaccine encoding the full-length S protein displayed advantage in post-immunized SARS-CoV-2 challenge test that the viral loads in bronchoalveolar lavage and nasal mucosa reduced significantly in post-immunized SARS-CoV-2 challenge test compared with the controls. The protective efficacy is positively related with the neutralizing antibody titers [151]. Erasmus et al. designed an Alphavirus-derived replicon RNA vaccine encoding the S protein of SARS-CoV-2, formulating with lipid inorganic nanoparticles, namely repRNA-CoV2S. A single dose of intramuscular injection of the vaccine candidate induced powerful secretion of anti–SARS-CoV-2 S protein IgG antibody which lasted as long as 70 days. Compared to young mice, aged mice may need to be immunized twice to enhance immune responses. This vaccine candidate will enter clinical trials under the name HDT-301 [152]. Further, the mRNA vaccine (mRNA-1273) expressing SARS-CoV-2 S protein produced by The Moderna platform was enclosed with lipid nanoparticles to promote entry of mRNA into host cells [153]. mRNA-1273 displayed promising immunological protective effects on mouse and nonhuman primate models [154, 155]. The results of the phase I, dose-escalation, open-label clinical trial enrolled 45 participants who received twice vaccine showed that the 100μg dose was more favorable with high neutralizing antibody titers and CD4 T cell response, no serious safety concerns. The phase 2 and phase 3 trials are ongoing [156]. Mark J et al. reported the study of a phase 1/2 trial of COVID-19 RNA vaccine BNT162b1 in 45 adults. They concluded that RBD-binding IgG concentrations and SARS-CoV-2 neutralizing titers were higher with higher dose and after a second vaccination. The geometric mean neutralizing titers were 1.9-6.4 times than that of COVID-19 convalescent sera, suggesting a well-tolerated and immunogenic dose of 10μg to 30μg [157].

Recombinant protein vaccine is also known as engineered subunit vaccine, recombinant target antigen is prepared through an expressing system in vitro and then purified for further use. E. Kim et al. developed microneedle arrays (MNAs) delivered SARS-CoV-2 S1 subunit vaccines based on their evolving experience with MNAs delivered MERS-S1 subunit vaccines due to that MAN delivery leads to high concentration of vaccine in the local skin microenvironment, promoting the efficacy of immunization. The results demonstrated that both MERS-S1 subunit vaccine and SARS-CoV-2-S1 subunit vaccine elicited significant and long-lasting specific antibody responses in C57BL/6 mice, supporting the development of relevant vaccines for clinical trials. In addition, advantages of MNA delivery compared to traditional needle injection have also been proven [158]. Yang et al. designed a recombinant protein vaccine targeting the RBD of S protein of SARS-CoV-2, which has been proven to be safe and effective to elicit protective immunity in three animal models, rodents, rabbits and non-human primates (macaca mulatta). Specifically, the vaccine introduced the RBD gene into Spodoptera frugiperda (Sf9) cells to produce high quality recombinant proteins, which were then refined and purified. This vaccine is easy to produce in large quantities. Additionally, a phase I clinical trial based on Sf9 cells has been launched in late August, 2020 [159].

Moreover, there are several ongoing clinical trials assessing the protective role of Bacillus Calmette–Guérin (BCG) vaccine against COVID-19 [160]. The mechanisms of BCG anti-COVID-19 consist in its non-specific effect on immune system (NSEs). NSEs enable to provide non-specific immunological protections against infections other than the original target disease. Briefly, The NSEs of BCG are mediated by the enhancement of innate immune response through epigenetic mechanisms, which are termed as “trained immunity” as well. This enhancing innate response have also been discovered in measles vaccine, oral polio vaccine and smallpox vaccine [161]. However, this nonspecific protection doesn’t last for a long time and decreases soon after the stimulus of BCG is removed from the body [162]. Although the epidemiological link between BCG and COVID-19 is remarkable [163], no statistically significant differences in the positive rate of SARS-CoV-2 infection between BCG-vaccinated population and the unvaccinated population, namely 11.7% and 10.4%, respectively [164]. WHO indicated that there is no evidence to date that BCG vaccine can protect people from SARS-CoV-2 infection. Therefore, BCG vaccination is not recommended to prevent COVID-19 at present [165]. Here, we also summarized the ongoing and future clinical trials of COVID-19 vaccine in Table 1.

**Table 1:** Ongoing and future clinical trials of COVID-19 vaccines

Type	NCT number	Vaccine/Platform	Phase	Estimated enrollment (status)
Inactivated vaccine	NCT04412538	West China Second Hospital, Sichuan university	Phase 1/2	942 (recruiting)
	NCT04352608	Sinovac Biotech Co., Ltd	Phase 1/2	744 (recruiting)
	NCT04383574	Sinovac Biotech Co., Ltd	Phase 1/2	422 (not yet recruiting)
Adenovirus vector vaccine	NCT04313127	Cansino Biologics Inc.	Phase 1	108 (active, not recruiting)
	NCT04398147	Cansino Biologics Inc.	Phase 1/2	696 (not yet recruiting)
	NCT04324606	ChAdOx1 University of Oxford	Phase 1/2	1090 (active, not recruiting)
	NCT04400838	ChAdOx1 University of Oxford	Phase 2/3	10260 (not yet recruiting)
	NCT04341389	Academy of Military Medical Sciences, PLA of China	Phase 2	508 (active, not recruiting)
Lentiviral vector vaccine	NCT04299724	Shenzhen Geno-Immune Medical Institute	Phase 1	100 (recruiting)
	NCT04276896	Shenzhen Geno-Immune Medical Institute	Phase 1/2	100 (recruiting)
mRNA vaccine	NCT04283461	mRNA-1273 National Institute of Allergy and Infectious Diseases (NIAID)	Phase 1	120 (active)
	NCT04405076	mRNA-1273 Moderna TX, Inc.	Phase 2	600 (active)
	NCT04380701	BNT162a1,BNT162b1,BNT162b2 ,BNT162c2 Biontech SE	Phase 1/2	200 (recruiting)
	NCT04368728	BNT162a1,BNT162b1,BNT162b2 ,BNT162c2 Biontech SE	Phase 1/2	7600 (recruiting)
DNA vaccine	NCT04336410	INO-4800 Inovio Pharmaceuticals	Phase 1	40 (recruiting)
Recombinant subunit vaccine	NCT04368988	Novavax	Phase 1	131 (recruiting)
	NCT04334980	Symvivo Corporation	Phase 1	84 (not yet recruiting)
	NCT04405908	SCB-2019 Clover Biopharmaceuticals AUS Pty Ltd	Phase 1	150 (Not yet recruiting)
Bacillus Calmette-Guérin (BCG) vaccine	NCT04348370	Texas A&M University	Phase 4	1800 (recruiting)
	NCT04369794	Leonardo Oliveira Reis, University of Campinas, Brazil	Phase 4	Not yet recruiting
	NCT04379336	TASK Applied Science	Phase 3	500 (recruiting)
	NCT04328441	MJM Bonten, UMC Utrecht	Phase 3	1500 (recruiting)
	NCT04327206	Murdoch Childrens Research Institute	Phase 3	10078 (recruiting)
	NCT04350931	Adel Khattab, Ain Shams University	Phase 3	900 ( Not yet recruiting)
	NCT04362124	Universidad de Antioquia	Phase 3	1000 (Not yet recruiting)
	NCT04384549	Assistance Publique - Hôpitaux de Paris	Phase 3	1120 (Not yet recruiting)
	NCT04387409	VPM1002 Vakzine Projekt Management GmbH	Phase 3	1200 (Not yet recruiting)
	NCT04373291	Bandim Health Project	Phase 3	1500 (Not yet recruiting)
Measles-Mumps-Rubella Vaccine	NCT04357028	Ahmed Mukhtar, Kasr El Aini Hospital	Phase 3	200 (Not yet recruiting)

In short, there are no approved human coronavirus vaccines at present. Though the first wave of the pandemic has been controlled in some countries, the number of infected people is increasing internationally. Therefore, the development of vaccine is still an urgent issue.

## Immunological treatment strategies against COVID-19

### Development of neutralizing antibodies

**Fig. 4:**
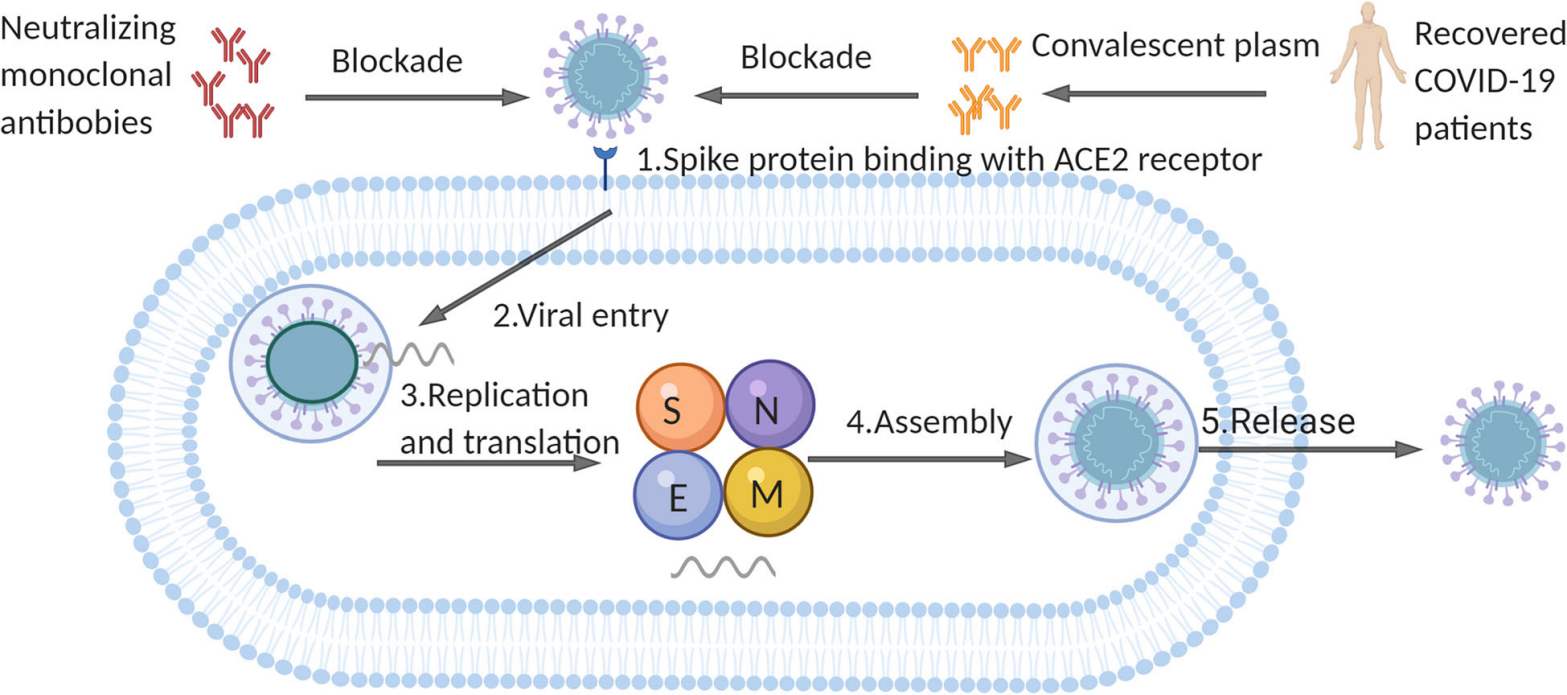
The hypothetic process of viral invasion into host cells and mechanisms of neutralizing antibodies. Binding of S protein and ACE2 receptor mediates the attachment of SARS-CoV-2 to host cells. After viral entry, the genomic RNA is released into cytoplasm to serve as a template. Then, large amounts of structural proteins S (spike protein), N (nucleocapsid protein), E (envelop protein), M (membrane protein) and genetic materials are synthesized through replication and translation. Finally, the assembled viruses are released to infect host cells nearby. Importantly, neutralizing antibodies existing in convalescent plasm show functions at blocking the adhesion of SARS-CoV-2 with ACE2 by binding the RBD region of S protein competitively. S: spike protein; N: nucleocapsid protein; E: envelop protein; M: membrane protein

Similar to SARS-CoV-1, SARS-CoV-2 invades the host cells expressing ACE2 receptors through its RBD located in the S1 subunit of S glycoprotein [166]. Therefore, specific NAbs exert efforts on interfering virus entry into cells by against ACE2 receptors or RBD domain (Fig. 4). First, some antibodies against SARS-CoV have been proposed due to its similarity with SARS-CoV-2. CR3022, SARS-CoV-1-specific human monoclonal antibody, was found to be useful against SARS-COV-2 by binding with its RBD. However, the most potent SARS-COV-specific NAb candidates (e.g. m396, CR3014) targeting the ACE2 binding site failed to bind with the S1 subunit of SARS-COV-2 [167]. CR3022 attributes to the cross-reactivity between SARS-COV and SARS-COV-2, but the targeting epitope of CR3022 is highly conserved and far away from the RBD site. Only two out of the three RBD domains of a S trimer were in the “up” confirmation and slightly rotated can the binding epitope be captured by CR3022 [168]. S309, a multiple monoclonal antibody recognizing SARS-CoV-2 S protein, was isolated from an individual infected by SARS-CoV-1 in 2003. The mechanism of S309 neutralizing activity lies in the recognition of a highly conserved glycan-containing epitope rather than competing with receptor binding [169]. Monoclonal antibody 47D11 was isolated from SARS-CoV infected transgenic mouse and neutralized SARS-CoV-2 (and SARS-CoV-1) in cell culture [170]. Subsequently, human-origin monoclonal antibodies from convalescent patients have been identified as well. Bin Ju et al. isolated and characterized 206 RBD-specific monoclonal antibodies from single B cells of 8 COVID-19 patients. The neutralizing efficacy of antibodies was related with their competitive power with ACE2 for RBD binding. It was the increase of steric hindrance of RBD elicited by NAbs that inhibits viral attachment with ACE2 and thereby blocks viral invasion [171]. B38 and H4 were able to block the binding of RBD with ACE2 because of their different epitopes on RBD, which most residues within B38-RBD complex overlap with RBD-ACE2 interface. Treatment assay of mouse model with the antibodies showed the reduction of viral loads [172]. Similarly, another potential virus-targeting MAb-pair CA1 and CB6 were disclosed, and CB6 was proved to protect rhesus monkeys from SARS-CoV-2 infection in both prophylactic and treatment conditions [173]. Cao et al. indicated a potent neutralizing antibody by high-throughput single B cell sequencing, BD-368-2, whose epitope was overlapped with the ACE2 binding site. BD-368-2 displayed satisfactory therapeutic and prophylactic efficacy on hACE2-transgenic mice with SARS-CoV-2 infection [174]. Cheng et al. cloned two human NAbs targeting SARS-CoV-2 RBD using memory B cells from recovered patients, namely 311mab-31B5 and 311mab-32D4. These two antibodies efficiently neutralize the S protein of pseudotyped SARS-CoV-2 [175].

### Convalescent plasma treatment

Convalescent plasma (CP) treatment, a classic passive immunotherapy, has been recommended by the National Health Commission of China in “ Diagnosis and treatment of COVID-19 (trial edition 6)” [176]. Experiences from previous epidemics, including SARS [177], H1N1 [178] and Ebola virus [179, 180], indicated that convalescent plasma therapy can significantly reduce the relative mortality of patients, which may be due to the inhibition of viremia by antibodies in convalescent plasma. Both humoral and cellular immune response were detected in most COVID-19 convalescent patients. The NAbs tiers are positively related with counts of virus-specific T cells [56]. Zhang et al. described retrospectively four critically ill patients with SARS-CoV-2 infection who received 200-2400 ml of CP from the 11th day to the 18th day of admission. All of them (including a pregnant woman) recovered from the disease at last [181]. Duan et al. observed the changes of clinical symptoms and laboratory parameters of 10 severely ill COVID-19 patients who received a 200 ml transfusion of CP with the neutralizing antibody titers over 1:640. The results showed that the 200 ml plasm transfusion were well tolerated. By 3 days post-transfusion, with the increase of saturation of oxyhemoglobin and lymphocyte counts, the decrease of CRP, the clinical symptoms were significantly improved. By 7 days post-transfusion, the viral load was undetectable in 7 patients who had viremia pre-transfusion [182]. Salazar et al. enrolled 25 critically ill COVID-19 patients and transfused them with 300 ml of CP. 14 days after transfusion, 19 patients (76%) had improved or been discharged [183]. Shen et al. compared the clinical outcomes before and after CP transfusion among 5 extremely ill patients. CP was offered 10-22 days after admission and the neutralization titer of antibodies was greater than 40. After CP transfusion, the clinical symptom improved largely with normalized body temperature, decreased Sequential Organ Failure Assessment (SOFA) score and viral load, increased oxygenation index (PaO_2_/FiO_2_) and serum antibody titer. 3 out 5 were discharged and the left were in stable condition. Importantly, Li et al. conducted the first randomized clinical trial performed in 7 medical centers in Wuhan, China, which contained 103 patients diagnosed with critical illness. The patients were divided into two groups. One group received CP and standard treatment together and the other group received standard treatment alone. The results showed no statistically significant clinical improvement or mortality within 28 days between the two group, but did provide an important signal of the anti-viral effect of high titer antibodies for an obviously negative conversion rate of viral PCR in CP treatment group compared with standard treatment group [184]. In addition, Zeng et al. treated 6 patients of end stage of COVID-19 with CP at a median of 21.5 days after viral shedding. The study suggested that administration of CP at the late stage was not effective and can’t help to reduce mortality [185].

Of note, though the therapeutic outcomes of some studies are optimistic, the optimal dose, administration time and the exact clinical benefits of CP treatment needed to be further studied in larger randomized controlled clinical trials. Also, antibody-dependent enhancement (ADE) is another concern. When non- NAbs produced and bound with virus, then the combination of Fc*γ* of antibodies with Fc*γ* receptors facilitated the virus invasion into host cells with Fc*γ* receptors, such as macrophages [186], intestinal epithelial cells [187] and kidney cells [188], leading to the amplification of viral replication [189]. ADE has been proved to occur among the infections of Dengue virus [190], Ebola virus [191] and SARS-CoV-1 [192]. ADE has also been proposed to explain the discrepancy in severity of the COVID-19 cases observed between China and elsewhere in the world [193]. Whether there is a role for ADE in COVID-19 still remains unknown currently, but attention must be paid both in CP treatment and the design of vaccination.

### Strategies for dampening CRS

In severe patients with SARS-CoV-2 infection, CRS is an important factor for disease aggravation and the major cause of ARDS and multiple-organ failure [83, 194]. At present, the proportion of COVID-19 patients enduring ARDS have been described to be up to 20%, which correlates with critically ill condition and severe outcomes [69, 101]. So, effective inhibition of the cytokine storm provides a promising strategy to reverse deterioration and save patients’ lives. Plenty of potential therapies targeting the host hyperinflammation are under exploration including blockade of inflammatory cytokines (IL-6, IL-1, IFN-*γ*), chloroquine (CQ), corticosteroid, blood purification treatments etc. among which IL-6 blockade is of great expectation given its key role in inducing CRS (Fig. 2) [195]. A number of clinical trials are undergoing at present to test the strategies of dampening CRS in SARS-CoV-2 infection (Table 2).

**Table 2:** Ongoing clinical trials of immune-related therapies for COVID-19

Therapy	Target	Mechanism	Status for COVID-19
Pembrolizumab	PD-1	Prevent T cell exhaustion	Phase 2: NCT04335305
Nivolumab	PD-1	Prevent T cell exhaustion	Phase 2: NCT04333914, NCT04356508, NCT04413838, NCT04343144
AMY-101	Complement C3	C3 inhibitor	Phase 2: NCT04395456
Zilucoplan	Complement C5	C5 inhibitor	Phase 2: NCT04382755, NCT04025632, NCT03225287, NCT03078582, NCT03030183 , NCT03315130
			Phase 3: NCT04225871, NCT04115293, NCT04297683,
Eculizumab	Complement C5	C5 inhibitor	Phase 2: NCT04346797
			Others: NCT04288713, NCT04355494
Tocilizumab	IL-6R	Recombinant humanized monoclonal antibody against IL-6R	Monotherapy:
			Phase 2: NCT04377659, NCT04370834, NCT04363853……
			Phase 3: NCT04403685, NCT04377503, NCT04361552……
			Phase 4: NCT04377750, ChiCTR2000029765,
			Others: NCT04332913,
			Combined therapy:
			+ Remdesivir: NCT04409262 (phase 3),
			+ Favipiravir: NCT04310228, ChiCTR2000030894,
			+ Azithromycin + HCQ/ Mefloquine: NCT04347031 (phase3), NCT04332094 (phase 2)
Sarilumab	IL-6R	A monoclonal antibody against IL-6R	Phase1: NCT04386239,
			Phase 2: NCT04322773, NCT04357808, NCT04357860
			Phase 2/3:NCT04315298, NCT04341870,NCT04324073,
			Phase 3: NCT04327388, NCT04345289
			Phase 4: NCT02735707
Clazakizumab	IL-6	A monoclonal antibody against IL-6.	Phase 2: NCT04348500, NCT04381052, NCT04343989, NCT04363502, NCT04363502
Olokizumab	IL-6	A monoclonal antibody against IL-6	Phase 3: NCT04380519
Siltuximab	IL-6	A chimeric antibody against IL-6	Phase 2: NCT04329650; Phase 3: NCT04330638; Others: NCT04322188
Ulinastatin		A serine protease inhibitor with anti-IL-6 property	Phase 1/2: NCT04393311
Anakinra	IL-1*β*	Antagonist of IL-1*β*	Phase 2: NCT04339712, NCT04412291, NCT04366232, NCT04357366, NCT04341584, NCT04339712,
			Phase 3: NCT04330638, NCT04364009, NCT04362111, NCT04330638, NCT04324021
			Phase 4: NCT02735707
			Others: NCT04408326, NCT04362943
Canakinumab	IL-1	Inhibitor of IL-1	Phase 2: NCT04365153; Phase 3: NCT04362813; Others: NCT04348448
Emapalumab	IFN-*γ*	Anti-IFN-*γ* monoclonal antibody	Phase 3: NCT04324021
Mavrilimumab	GM-CSF	Inhibitor of GM-CSF	Phase 2: NCT04397497, NCT04399980
CQ or HCQ		Reduce pro-inflammatory cytokine levels	Phase 2: NCT04328493, NCT04344951, NCT04333914……
			Phase 2/3: NCT04333628, NCT04353336, NCT04351347, NCT04403555……
			Phase 3: NCT04360759, NCT04342221, NCT04371406……
			Pahse 4: NCT04362332, NCT04331600, NCT04286503, NCT04351191……
Corticosteroids		Non-specific cytokine suppression	Phase 2: NCT04344288, NCT04329650, NCT04360876……
			Phase 3: NCT04359511, NCT04345445, NCT04395105……
			Phase 4: NCT04355637, NCT04325061, ChiCTR2000029656……
Plasma exchange		Remove excessive cytokines in the blood	Phase 2: NCT04374539, NCT04374149
IVIG		Neutralization of cytokines	Phase 2: NCT04403269; Phase 2/3:NCT04261426;
			Phase 3: NCT04400058; Phase 4: NCT04411667
MSCs		Reduce the production of pro-inflammatory cytokines	Phase 1: NCT04252118, NCT04313322, NCT04302519……
			Phase1/2: NCT04336254, NCT04346368, NCT04366323……
			Phase 2: NCT04315987, NCT04416139, NCT04288102……
			Phase2/3: NCT04366063,
			Phase 3: NCT04371393,
IFN-I		Antiviral immune response	Phase 1: NCT04293887,
			Phase 2: NCT04343976, NCT04465695, NCT04343768……
			Phase 3: NCT04492475, NCT04320238, NCT04324463……
			Phase 4: NCT04350671, NCT04350684, NCT04291729……
IFN-III		Antiviral immune response	Phase 2: NCT04343976, NCT04354259, NCT04344600
Neutralizing antibodies	S protein	Blockade the attachment of RBD with ACE2	Phase1/2:NCT04354766
Convalescent Plasma		Acquirement of passive immunity	Phase1: NCT04333355, NCT04397757, NCT04388527…
			Phase1/2: NCT04384497, NCT04390178, NCT04356482…
			Phase2: NCT04415086, NCT04343755, NCT04389710…
			Phase2/3: NCT04388410, NCT04342182, NCT04374526…
			Phase3: NCT04418518, NCT04372979, NCT04348656…

#### Antagonists of IL-6 and IL-6R

Tocilizumab, a recombinant humanized monoclonal antibody against IL-6R, could firmly bind to soluble and membrane-bound IL-6R thus prevent the binding of IL-6 to IL-6R and block signal transduction [82]. Owing to its remarkable efficacy, tocilizumab was approved as a biological therapy of CAR T-cell treatment induced CRS in adults and children by FDA (in 2017) and EMA (in 2018) [91, 196–199]. Recently, tocilizumab was repurposed to be the key to reduce CRS-related mortality and its use has been added into the management guidelines of severe or critically ill COVID-19 patients with extensive lung lesions and high level of IL-6 in China [77, 196, 200, 201]. Researchers worldwide are working on to evaluate this drug as well [202]. The recommended dose of tocilizumab is 4-8mg/kg and once up to a maximum of 800mg intravenous drip at the first administration while no more than twice should the severe patients be given tocilizumab therapy.

Accumulating evidence showed that tocilizumab may be the key to reduce mortality in severe COVID-19 patients with CRS [203]. A case reported that two infusions of tocilizumab contributed to a rapidly improved outcome in a COVID-19 patients with respiratory failure [204]. Similarly, a small sample clinical trial in China (ChiCTR2000029765) involving 21 severe cases demonstrated that tocilizumab is capable of ameliorating high fever and lung lesion, as well as normalizing the decreased lymphocytes and increased CRP level of COVID-19 patients within 5 days of administration of a single 400 mg dose [82]. Later on, a single center study involving 100 severe COVID-19 patients with ARDS characterized with hyperinflammatory syndrome showed a significant and lasting response to tocilizumab treatment [205]. However, some of these researchers are uncontrolled non peer reviewed, and a subsequent level of IL-6 after treatment initiation was not included. These results are in urgent need of confirmation in more randomized controlled trials (RCTs). Currently, no less than 30 clinical trials are approved to test the efficacy and safety of tocilizumab for the treatment of pneumonia in COVID-19 as monotherapy or in combination with anti-virus drugs (remdesivir and favipiravir), azithromycin or anti-malaria drugs (hydroxychloroquine (HCQ) and mefloquine).

Apart from tocilizumab, other inhibitors of IL-6 signaling axis are also under intensive exploration for the treatment of SARS-CoV-2 infection, including sarilumab (a monoclonal antibody against IL-6R), siltuximab (a chimeric antibody of IL-6), clazakizumab and olokizumab (two humanized monoclonal antibodies against IL-6), among which sarilumab and siltuximab are FDA-approved agents against IL-6 [67, 206–208]. A phase II/III RCT (NCT04315298) enrolled 400 cases is ongoing to assess the efficacy and safety of sarilumab in hospitalized patients with COVID-19 compared with placebo. Sarilumab is also being tested for the therapy of moderate cases of SARS-CoV-2 infection (NCT04359901). Siltuximab is another therapeutic option to block the involvement of IL-6 in COVID-19 patients [209]. The efficacy and safety of siltuximab are being evaluated in comparison with the broad immunosuppressor methylprednisolone in hospitalized patients with COVID-19 pneumonia (NCT04329650). In addition, clazakizumab is administered more frequently to COVID-19 patients with life-threatening pulmonary failure associated with CRS (NCT04381052, NCT04343989, NCT04363502). A phase II/III double-blinded RCT is evaluating the efficacy of a single dose of 64mg olokizumab for the treatment of severe COVID-19 patients besides standard therapy (NCT04380519). Therefore, all patients with severe SARS-CoV-2 infection are supposed to be screened hyperinflammation biomarkers in order to pick out suitable cases that would benefit from immunosuppression therapy.

Thus far, reports detailing the patients’ outcomes receiving IL-6 blockade therapy were still insufficient. Despite the promising efficacy of IL-6/IL-6R blockers in the management of SARS-CoV-2 infection, we should notice that some side effects may also come along. One theoretical possibility is that immunosuppression as a result of IL-6 antagonism may contribute to delayed viral clearance, fungi infection and osteonecrosis of the jaw as observed in rheumatoid arthritis patients treated with these drugs [69]. Radbel et al. reported two cases of COVID-19 with CRS receiving tocilizumab treatment both progressed to sHLH and one developed viral myocarditis [210]. Decreased IL-6 may contribute to enhanced viral replication if tocilizumab is administered too early in the disease course [210]. Other common adverse reactions of IL-6 inhibition include increased serum transaminases and lipid concentrations, pancreatitis and so on [199]. Hence, the results of safety evaluation of IL-6 inhibition are in urgent need. We should also take efforts to determine the optimal patient selection and timing for the utility of anti-IL-6 therapy for COVID19-induced CRS.

Intriguingly, we can modulate CRS by indirectly interfering with the levels and functions of IL-6/IL-6R. One possible strategy is to inhibit JAK, an important downstream component of IL-6 pathway in CRS [77, 195, 211]. Baricitinib, tofacitinib, ruxolitinib are most intensively investigated JAK inhibitors for the treatment of SARS-CoV-2 infection (NCT04390061, NCT04377620, NCT04340232, etc.). CRS can also be prevented by blocking the catecholamine-cytokine axis which augments the production of IL-6 and other cytokines [67]. An open label RCT (NCT04365257) is undergoing to test prazosin, an *α*1-AR antagonist, on preventing CRS and severe complications in hospitalized COVID-19 patients. Ulinastatin, a serine protease inhibitor with anti-IL-6 capability, is another possible immunomodulatory agent of the cytokine storm [212]. Ulinastatin can reduce TNF-*α* and IFN-*γ* levels, as well as promoting the secreting of anti-inflammatory IL-10, which could assist the body to regain balance between pro-inflammatory and anti-inflammatory responses [213, 214]. The effect of ulinastatin is being evaluated on time to recovery, disease severity, need for ventilator support, and mortality of hospitalized COVID-19 patients compared to placebo in a multi-center randomized study at present (NCT04393311).

#### Inhibition of the IL-1 pathway

During CRS, the enhanced expression of IL-1 was observed to peak before the respiratory failure which indicated that IL-1 might drive the pathogenesis of COVID-19 [95]. Hence, inhibition of IL-1 pathway has attracted great attention. Anakinra (an antagonist of IL-1*β*) and canakinumab (IL-1 inhibitor), once utilized for the clinical management of severe sepsis, is now repurposed to fight against SARS-CoV-2 infection to prevent the respiratory dysfunction [95]. However, the clinical experience of applying these agents to treat COVID-19 is far from sufficient. Therefore, animal experiments and clinical trials are necessary to determine their effects and safety. Correspondingly, plenty of trials are now being launched to test the efficacy of anakinra (NCT02735707, NCT04330638, NCT04364009…) and canakinumab (NCT04365153, NCT04362813, NCT04348448) for COVID-19 patients. Routine examination of the levels of inflammatory cytokines is required to monitor these agents’ influence.

#### Inhibition of other cytokines

The occurrence of CRS attributes to the increase in various cytokines. Therefore, inhibition of other increased cytokine, including IFN-*γ* and GM-CSF, is also a possible method to manage severe COVID-19 patients [83]. Using emapalumab, a monoclonal antibody against IFN-*γ*, may be able to reduce the numbers of severe COVID-19 patients requiring mechanical ventilation and ICU care, as well as reduce the mortality (NCT04324021). Inhibition of GM-CSF provides another perspective as described in two newly launched clinical trials (NCT04397497 and NCT04399980).

#### IFN-I and IFN-III

Administration of IFN-I and IFN-III in COVID-19 has been an issue of heated debate. Utilization of nebulized IFN-*α*2b alone or in combination with arbidol decreased the duration of detectable virus and inflammatory markers, as reported in a retrospective study [215]. Vapor inhalation of IFN-*α* in combination with ribavirin has been added into China management guidelines of COVID-19 [216]. *In vitro* studies demonstrated that SARS-CoV-2 was sensitive to IFN-I supplement [98, 100]. Ivan et al. reported the results of an open-label, randomized, phase 2 clinical trial (NCT04276688) that assessed the efficacy of a triple combination regimen of IFN-*β*1b, lopinavir-ritonavir, and ribavirin in the treatment of 127 hospitalized COVID-19 patients [217]. Triple therapy significantly shortened the duration of viral shedding, symptom alleviation, and hospital stay compared with control group. To further explore the potency of IFN-I and IFN-III for COVID-19 management, various types of IFN are being evaluated in clinical trials, including IFN-I type: IFN-*α*1b (NCT04320238), IFN-*α*2b (NCT04480138), IFN-*β*1a (NCT04350671), IFN-β*1*b (NCT04465695), and IFN-III type: IFN-*λ* (NCT04343976), IFN-*λ*1a (NCT04354259).

#### Chloroquine and hydroxychloroquine

CQ and its derivative HCQ have been used against SARS-CoV-2 infection in patients aged 18-65 years [83, 212]. Previously, CQ phosphate was administered for the treatment of autoimmune diseases because of its anti-inflammatory properties, including inhibiting the expression of TNF, IL-6 and major histocompatibility complex class-II (MHC-II) [218, 219]. Moreover, CQ and HCQ are weak bases that can accumulate and increase the pH in endosome and lysosome, thus inhibiting viral replication [220]. CQ and its derivatives have shown apparent efficacy and acceptable safety against SARS-CoV-2 induced pneumonia in plenty of trials with or without azithromycin [218, 221]. The preferred dose for the treatment of COVID-19 is as follows: CQ: 500 mg, twice per day for patients more than 50Kg and 500 mg, twice per day on the first two days and once per day on the following 5 days for patients less than 50Kg; HCQ sulphate: 200 mg, three times per day [212]. However, current data on the benefits and harms of HCQ or CQ for COVID-19 treatment is far from clear and often contradictory, as reported in a systematic review conducted by Adrian and colleagues [222]. A high dose of CQ for more than 600mg may pose the patients into a death threat [223].

In order to draw a conclusion upon whether CQ are beneficial for COVID-19 patients or not, dozens of clinical trials are carrying out at present alone or in combination with azithromycin. A multicenter, open label RCT is trying to evaluate if adding CQ or HCQ to only supportive care may ameliorate disease progression in hospitalized moderate to severe COVID-19 patients (NCT04362332). Similarly, a phase 2b study is undergoing to test the efficacy and safety of CQ on hospitalized COVID-19 patients, as well as measure whether CQ is capable of reducing mortality after 28-day follow-up (NCT04323527). A phase 3 RCT including 2770 participants is working on to demonstrate the efficacy of CQ combined with azithromycin on early stage disease in COVID-19 patients (NCT04371406). Since the convincing evidence in support of using CQ or HCQ as a therapeutic option for SARS-CoV-2 infection is insufficient, the results of these ongoing clinical trials is in unprecedented need.

#### Corticosteroids

Corticosteroids are widely used as an immunosuppressor to inhibit CRS [74]. However, whether COVID-19 patients would benefit from corticosteroids treatment is of great debate. Some considered that corticosteroids would induce host immune suppression and delay viral clearance, while others consented that corticosteroids are strong inhibitors of the hyperinflammatory state which is the main cause of death in severe COVID-19 patients [224, 225]. Use of corticosteroids-based therapy in severe and critically ill patients has been reported previously [6, 12]. Some studies indicated that treatment with corticosteroids reduced the risk of death, especially in patients with ARDS, instead of affecting viral clearance time and prolonging duration of symptoms or hospital stay [226, 227]. However, based on the experiences of using corticosteroids in other viral pneumonia like SARS, improper use of systemic corticosteroids may cause serious side effects including osteonecrosis of the femoral head. Therefore, corticosteroids should be used with caution for certain severe cases of COVID-19 suffering from CRS, not in mild cases. Routine use is not supported [228, 229]. Of note, corticosteroids should only be used at low-to-moderate doses for a short term (3-5 days) [212].

A phase 3 study is evaluating the efficacy and safety of prednisone and hydrocortisone on treating oxygen-dependent COVID-19 patients with ARDS (NCT04359511). Another open label phase 4 RCT is testing whether inhaled budesonide could reduce treatment failure in COVID19 patients with pneumonia (NCT04355637). Other corticosteroids including methylprednisolone and different administration routines (like budesonide dry powder inhaler, dexamethasone injection) are under investigation for the treatment of SARS-CoV-2 infection (NCT04345445, NCT04416399, NCT04360876……).

#### Blood purification treatments

Another approach to alleviate CRS is blood purification treatments which can fundamentally remove the excessive inflammatory cytokines in the blood. This purification system includes multiple steps like plasma exchange, adsorption, perfusion, etc. [83] Zhang et al. introduced an artificial-liver blood-purification system which is applied in Zhejiang province and presented a good efficacy for the treatment of patients with serious SARS-CoV-2 infection [230]. At present, 2 clinical trials are undergoing to evaluate the efficacy of plasma change alone or in combination with ruxolitinib (a JAK/STAT pathway inhibitor) in severe COVID-19 patients with CRS requiring invasive mechanical ventilation (NCT04374539, NCT04374149).

#### Intravenous immunoglobulin (IVIG)

In a retrospective study carried out by Chen et al. [6], about 27% of COVID-19 patients were given IVIG treatment [6]. The recommended dose of IVIG to interrupt CRS is 0.3-0.5g/Kg per day for 5 days [50]. Octagam, an IVIG agents, are being evaluated to modulate the immune system, and stabilize or improve clinical outcomes of severe COVID-19 patients (NCT04400058, NCT04411667). Another RCT of IVIG therapy for severe SARS-CoV-2 infected pneumonia has been launched (NCT 04261426). Nevertheless, current evidence is sparse about the efficacy of IVIG therapy on COVID-19 patients.

#### Mesenchymal stem cells (MSCs)

As an essential member of stem cell family, MSCs exerted unique properties of inhibiting inflammation, modulating immune response and repairing damaged lung tissue [8]. On the one hand, MSCs could ameliorate CRS by inhibiting T cells and macrophages and reduce the production of different kinds of pro-inflammatory cytokines. On the other hand, MSCs could promote the generation of Treg cells and secrete inhibitory cytokine IL-10, alleviating ARDS [83]. Those properties made MSCs a promising method for the therapy of COVID-19. IFN-*γ* activated MSCs may be more effective in the inhibition of hyperimmune response [9]. A phase 3 clinical trial is ongoing to evaluate the efficacy and safety MSCs in COVID-19 patients with ARDS compared with placebo (NCT04371393). Similarly, MSCs alone or plus extracellular vesicles against SARS-CoV-2 infection with ARDS is assessed in comparison to conventional therapy and supportive care (NCT04366063). Other current clinical trials of MSCs for severe COVID-19 patients mainly reached early phase 1 and phase 2 (NCT04252118, NCT04336254, NCT04315987……).

## Conclusion

The sudden outbreak and rapid spread of SARS-CoV-2 mounted a significant challenge to the global health systems. Although we knew nothing about its characteristics from the very start, previous studies based on SARS-CoV-1 and MERS-CoV accelerated our understanding of this novel coronavirus in a short period of time. However, neither vaccines nor specific anti-SARS-CoV-2 drugs are available currently. Next, compared to other coronavirus infections, there still are many differences in immune response to SARS-CoV-2 infection according to the existing proof. Finally, it should be noticed that the immune system does play critical roles in fighting viruses, but the side effects such as CRS and ADE are also harmful. Therefore, it is imperative to further elucidate the underlying immunological mechanisms in disease pathogenesis and progression for better design of diagnostic, therapeutic and preventive strategies of COVID-19.

## Data Availability

Not applicable

## Abbreviations

ACE2: Angiotensin converting enzyme II
ADE: Antibody-dependent enhancement
AngII: Angiotensin 2
ARDS: Acute respiratory distress syndrome
AT1R: Angiotensin receptor type 1
AUC: Area under curve
BCG: Bacillus Calmette–Guérin
CLIA: Chemiluminescence immunoassay analysis
COVID-19: Coronavirus disease 2019
CP: Convalescent plasma
CQ: Chloroquine
CRP: C-reactive protein
CRS: Cytokine release syndrome
CTLA-4: Cytotoxic T lymphocyte antigen 4
CTLs: Cytotoxic CD8+ T cells
DEGs: Differentially expressed genes
ELISA: Enzyme-linked immunosorbent assay
G-CSF: Granulocyte-colony stimulating factor
GICA: Colloidal gold immunochromatographic assay
GM-CSF: Granulocyte-macrophage colony-stimulating factor
HCQ: Hydroxychloroquine
IC: Immunochromatographic
ICU: Intensive care unit
IFN-III: Type-III IFN
IFN-I: Type-I IFN
IFN-γ: Interferon-γ
IL: Interleukin
IP10: IFN-γ inducible protein 10
ISGs: IFN-stimulated genes
JAK: Janus kinase
MAPK: Mitogen-activated protein (MAP) kinase
MCLIA: Magnetic chemiluminescence enzyme immunoassay
MCP1: Monocyte chemoattractant protein 1
MDR: Multiple organ failure
mIL-6R: trans-membrane IL-6 receptor
MIP1β: Macrophage inflammatory protein 1β
MNAs: Microneedle arrays
N protein: Nucleocapsid protein
N8R: Neutrophil-to-CD8+ T cell ratio
NAbs: Neutralizing antibodies
Nabs: Neutralizing antibodies
NETs: Neutrophil extracellular traps
NK: Natural killer
NLR: Neutrophil-to-lymphocyte ratio
NSEs: Non-specific effect on immune system
PD-1: Programmed death-1
RBD: Receptor binding domain
RCT: Randomized controlled trials
RT-qPCR: Real time reverse transcriptase quantitative polymerase chain reaction
PCR: Polymerase chain reaction
S protein: Spike protein
SARS-COV-2: Severe acute respiratory syndrome coronavirus 2
Sf: Spodoptera frugiperda
sHLH: secondary hemophagocytic lymphohistiocytosis
sIL-6R: soluble IL-6 receptor
SOFA: Sequential Organ Failure Assessment
STAT3: Signal transducer and activator of transcription 3
Th: Helper T
TNF-α: Tumor necrosis factor-α
Treg: Regulatory T
VEGF: Vascular endothelial growth factor
WBC: White blood cell
